# Word-Decoding as a Function of Temporal Processing in the Visual System

**DOI:** 10.1371/journal.pone.0084010

**Published:** 2013-12-20

**Authors:** Steven R. Holloway, José E. Náñez, Aaron R. Seitz

**Affiliations:** 1 Department of Psychology, Arizona State University, Tempe, Arizona, United States of America; 2 Department of Social and Behavioral Sciences, Arizona State University, Phoenix, Arizona, United States of America; 3 Department of Psychology, University of California – Riverside, Riverside, California, United States of America; University of Leicester, United Kingdom

## Abstract

This study explored the relation between visual processing and word-decoding ability in a normal reading population. Forty participants were recruited at Arizona State University. Flicker fusion thresholds were assessed with an optical chopper using the method of limits by a 1-deg diameter green (543 nm) test field. Word decoding was measured using reading-word and nonsense-word decoding tests. A non-linguistic decoding measure was obtained using a computer program that consisted of Landolt C targets randomly presented in four cardinal orientations, at 3-radial distances from a focus point, for eight compass points, in a circular pattern. Participants responded by pressing the arrow key on the keyboard that matched the direction the target was facing. The results show a strong correlation between critical flicker fusion thresholds and scores on the reading-word, nonsense-word, and non-linguistic decoding measures. The data suggests that the functional elements of the visual system involved with temporal modulation and spatial processing may affect the ease with which people read.

## Introduction

Many theories, causal and non-causal, have been advanced in an effort to explain the frequent co-occurrence of diminished dorsal stream function and linguistic deficits. Although the precise role remains unclear, it is generally accepted that some visual system deficits are associated with reading impairments such as dyslexia [1], [2]. One such measure that has garnered considerable attention is the Critical Flicker Fusion (CFF) threshold. CFF thresholds have also been shown to be impaired in populations with reading disorders [3], [4]. While CFF and reading scores have been compared between normal reading and impaired populations, few studies, if any, have compared CFF thresholds and decoding abilities within a normal reading population.

There is substantial evidence supporting the relationship between CFF thresholds and cortical processing capacity. For example, lesion studies in non-human primates indicate that processing in the magnocellular visual pathway [5], [6] and occipital lobe [7], [8] are rate-limiting for CFF. Likewise, most neuroscience research in animals points towards flicker fusion being largely mediated by cells in the dorsal visual pathway which are specialized to process high temporal frequencies, respond to low-luminance contrasts, and are involved in motion processing [9]–[14].

The dorsal visual stream is also thought to be affected in some individuals with reading disabilities. Graves, Frerichs, and Cook [15] found that people who suffered from a reading disability or dyslexia demonstrated a deficit in reporting the locations of small targets of varying contrasts - a task also known to be mediated in the dorsal stream of the visual cortex. Cornelissen, Richardson, Mason, Fowler, and Stein [16] found that dyslexics, as compared to a control group, were significantly less sensitive to motion under varying levels of contrast. Moreover, Demb, Boynton, and Heeger [17] reported that dyslexics showed reduced brain activity in the primary visual cortex, specifically in area V1, as well as several extrastriate areas, including MT and MT+. Processing written language and the difficulties in visual perception of speed and motion may stem from inefficiency of, or damage to, the transient dorsal stream of the visual system which includes the magnocellular pathway and extrastriate cortex such as area V5/MT [18]–[23].

While there is a fair amount of evidence that supports a link between CFF and reading disabilities [24], there is little evidence in the normal population of the link between CFF and reading, particularly, reading as measured by decoding ability. As a step toward understanding this relationship, the present study was designed to investigate the possible relation between visual temporal processing and reading as measured by word decoding, non-word decoding, as well as non-linguistic decoding ability, in a normal reading population.

## Methods

### Participants

Informed consent was obtained in writing from all participants, and this study conformed to the tenants of the Declaration of Helsinki. The Arizona State University Office of Research Integrity and Assurance specifically approved this study. Forty participants (32 females), ages 18–31 years, were recruited from Arizona State University through an Introduction to Psychology participant pool and were offered extra credit for their participation. Demographics were collected through a questionnaire, and all participants self-reported average to above average reading ability. (While it would have been preferable to use a standardized reading-assessment, the fact that the students were successfully enrolled in university-level course work involving a great amount of reading materials supports use of the self-report method.) All participants had normal or corrected-to-normal vision (measured on-site with a Snellen chart and protocol) and were naive as to the purpose of the experiment. The non-linguistic decoding task took participants an extra 45 minutes to an hour to complete, and only a subset (18; 16 females) of these subjects could participate due to the extra time commitment. All CFF, word decoding, and non-linguistic decoding data was collected by the same experimenter.

### Measures and Procedure

Critical flicker fusion thresholds were assessed with a Terahertz Technologies C-995 optical chopper using the method of limits (the mean of three descending measures from a high speed of flicker to a low speed in which the participants reported when the stimulus begins to flicker and three ascending measures from a slow speed to a fast speed in which the participant reported when the flicker stops) by a 1-deg diameter green (543 nm) test field.

After CFF thresholds were established, participants were asked to quickly read, out-loud, a list of nonsense words, organized in columns, which increased in reading difficulty; then, they were asked to do the same with a word decoding test. The word decoding task used a modified version of the La Pray & Ross [25] San Diego Quick Assessment (SDQA) reading-word decoding test (see Table 1) and a SDQA related nonsense-word decoding test (see Table 2). We were specifically targeting decoding errors that were likely related to reading difficulties similar to the errors made by dyslexics. So, unlike the standard use of the San Diego measure, decoding errors were assigned when a subject pronounced a letter as if it were inverted or, more commonly, when switching letter or syllable sounds within a word (e.g., “prevalence” pronounced as “pre**l**a**v**ence”). We did not measure reading ability in the participants, only decoding. The modified measure was employed in an effort to isolate the aspects of reading that were most likely to be related to dorsal stream function. This was important especially considering the participants were drawn from a population where bilinguals often pronounce certain letters differently than native English speakers, and this manner of general mispronunciation is unrelated to the question-at-hand. As with the standard San Diego Quick Assessment reading-word decoding test, the words were numbered from least to most difficult, and the number of the last word to be spoken before a decoding error was observed became the score given to the participant. As mentioned above, other pronunciation errors were not included in the scoring criteria. Moreover, the non-word decoding test was created by modifying the word list of the SDQA such that a comparable non-word measure could be utilized. In this way, we could directly assess if known words were processed differently than pronounceable non-words.

**Table 1:** Sample of the Word Decoding Test Sheet.

Word Decoding Sheet				
A	how	was		middle
B	see	city		several
O	cat	letter		moment
P	milk	myself		believe
E	always	animal		weather
R	tree	early		carefully
T	bigger	himself		block
H	book	quietly		awake
[8]	[16]	[24]		[32]
size	severed	business		contemporary
board	amazed	residence		commercial
frightened	improved	quarantine		threshold
exclaimed	quality	contagious		participate
trickle	escape	glutton		apparatus
approve	certainly	exhaust		desolate
lonely	interrupted	squirming		eliminate
stalker	grieve	acquainted		triumph
[40]	[48]	[56]		[64]
tranquility	emphasis	capacious		anomaly
humidity	condescend	prevalence		conscientious
contemptuous	rescinded	repugnant		vulnerable
impetuous	luxurious	peculiarity		deteriorate
humiliate	unanimous	rudimentary		spurious
conspiracy	intrigue	pugilist		irascible
aeronautic	protuberance	mitosis		expunge
predilection	audacious	molecule		coercion
[72]	[80]	[88]		[96]
discretionary	oligarchy	pseudonym		longevity
enigmatic	exigencies	rotunda		residual
prevaricate	mnemonic	idiosyncrasy		vehemence
centrifugal	ingratiating	exonerate		regicidal
itinerary	covetousness	misogyny		evanescence
abysmal	aborigines	desuetude		heinous
soliloquize	emaciated	exophthalmic		omniscience
gratuitous	seismograph	succinct		superannuate
[104]	[112]	[120]		[128]

**Table 2:** Sample of the Nonsense-word Decoding Test Sheet.

Nonsense-word Decoding Sheet			
A	hol	ras	wibble
B	sle	cimy	sekral
O	gar	liser	vodent
P	filk	mocelf	feliene
E	abweys	cynudal	wiacher
R	jree	eably	saredully
T	figger	hinseff	plock
H	beik	quably	ewaik
[8]	[16]	[24]	[32]
sidle	sweverd	bufelness	conhemborary
goarp	amaged	rekizence	jommerdial
frichrend	imploved	quajanmine	shrethold
exshained	quamity	lontagious	warvicitate
trinle	egcape	ghuttob	apparazus
appluve	mertainly	enhaubt	desotate
lokely	inreupted	cluirming	egimicate
ralker	srieve	acquaimsed	triunth
[40]	[48]	[56]	[64]
branquitity	elphasis	papacious	anovaly
hugimidy	contesen	trevequence	conthiensious
monlemptuous	yiscinded	depughant	vulderable
impebuous	buxurious	pebuliarity	dileriocate
shumipiate	unaminous	wunimendary	sirurious
quonhiracy	inbrigue	mugilist	irastibel
laeronaubic	croduberance	pitiosis	exfunjie
gredilection	audepious	mocetule	coerdion
[72]	[80]	[88]	[96]
disfretionady	olifarthy	psleugomym	gonglivity
enilpratic	exivenicies	fojunda	reliduam
prebarilate	snezonic	ipiomyndrasy	detemence
jentrilugal	ingrapliating	exonesate	medicival
ibinetary	kovelousness	misobyney	ebanesceilce
ubysmal	aboribines	mesuelude	heianous
soviloquite	ebaciager	exiphabalmic	omdistience
grabulitous	seislograte	subcinct	tuperalnuate
[104]	[112]	[120]	[128]

A psychophysical non-linguistic decoding measure was obtained from 18 of the 40 participants using a computer program that consisted of Landolt C targets randomly presented in four cardinal orientations, at 3-radial distances from a focus point, for eight compass points, in a circular pattern. Subjects responded by pressing the arrow key on the keyboard that matched the direction the target was facing. Percentage correct was assessed over five blocks of 96 trials each for a total of 480 trials. This psychophysical test is non-linguistic because it is more akin to novel shape recognition than it is to reading, yet, it still requires the visual system to assess the direction of the opening in a manner similar to word decoding. CFF thresholds were compared to word decoding, nonsense-word decoding, and non-linguistic decoding scores.

## Results

Performance on the word decoding task (M = 85.11, SD = 14.82), nonsense-word decoding task (M = 41.39, SD = 16.84) and the CFF thresholds (M = 22.42, SD = 1.58) varied significantly across participants. Scores on the word decoding test were comparable to the original San Diego Quick Assessment measure when transformed as the SDQA test requires (by dividing by ten) [26]. A key purpose of our study was to understand this variability in performance across measures.

**Figure 1:**
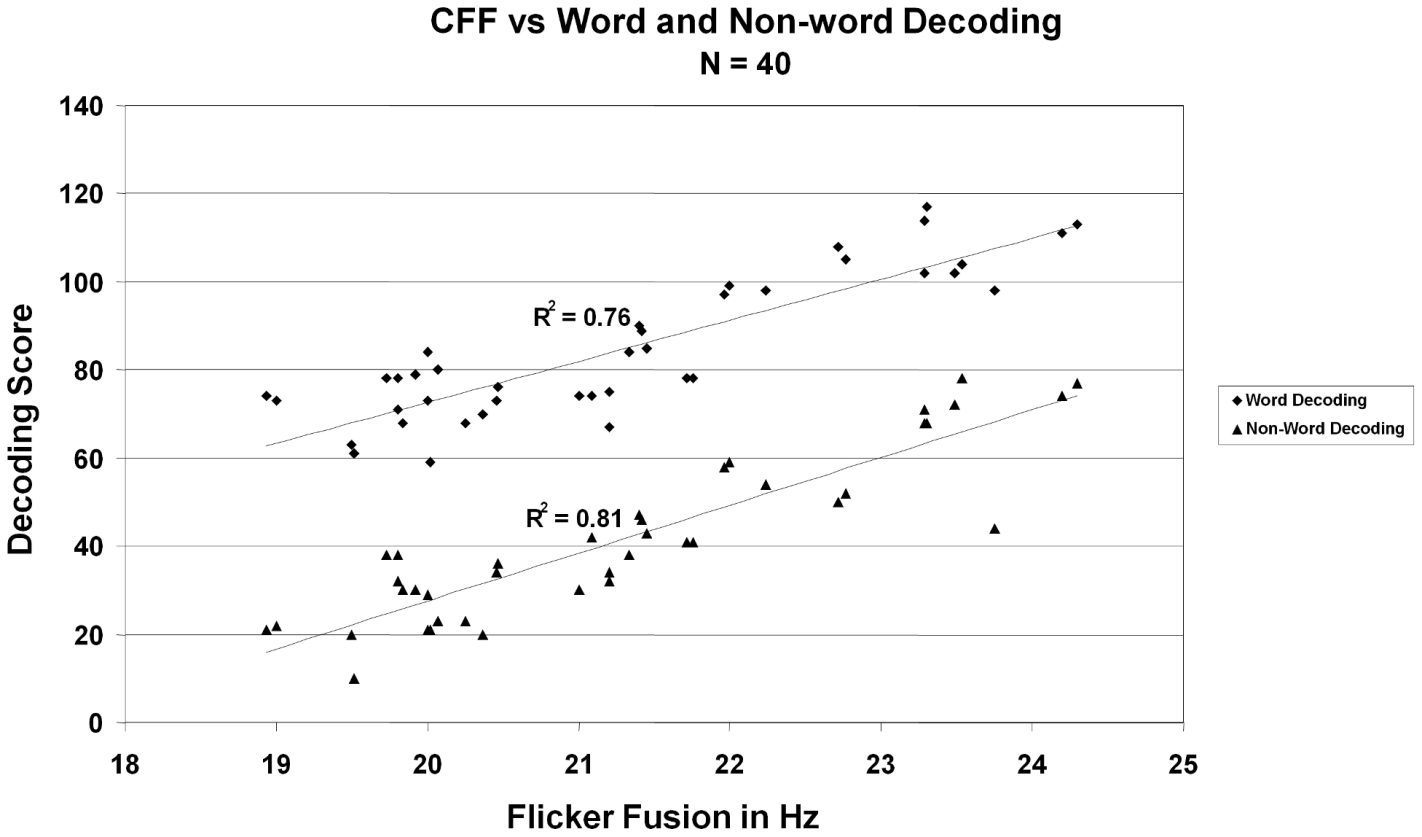
Correlation between CFF threshold and Word Decoding Test scores (F(39) = 125.46, r = .88, r^2 = ^.76, p<.01), and Nonsense-word Decoding Test scores (F(39) = 168.36, r = .90, r^2^ = .81, p<.01).

To assess the relationship between CFF thresholds and decoding, we examined the correlation between these two measures. Results demonstrated a very strong correlation between CFF thresholds and scores on the word decoding test F(39) = 125.46, r = .88, r^2^ = .76, p<.01, and between CFF thresholds and scores on the nonsense-word decoding test, F(39) = 168.36, r = .90, r^2^ = .81, p<.01 (see Figure 1).

**Figure 2:**
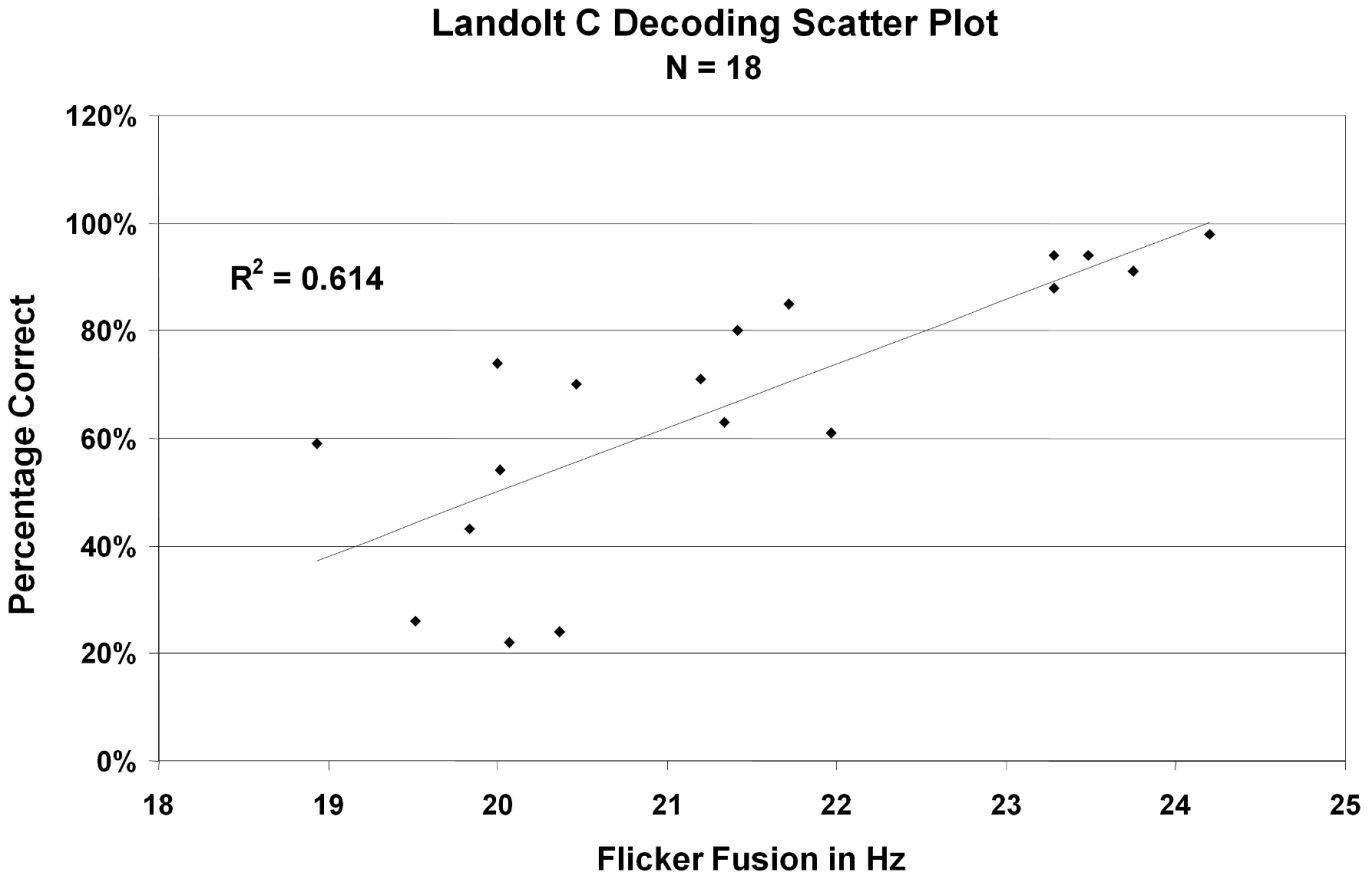
Correlation between CFF thresholds and Landolt C test scores (F(17) = 25.45, r = .78, r^2^ = .61, p<.01).

While these results demonstrate a strong relation between CFF thresholds and word decoding, the similar effect with words and non-words are suggestive of a non-linguistic origin of the effect. To test this, we ran a subset of the participants on the Landolt C task, with the goal of identifying whether the relationship may be related to processing the shapes of the individual letters. A strong correlation was also observed between CFF thresholds (M = 21.4, SD = 1.56) and scores on the non-linguistic Landolt C decoding test (M = .55, SD = 0.21), F(17) = 25.45, r = .78, r^2^ = .61, p<.01 (see Figure 2). These results suggest a deficit in visual processing that underlies word decoding abilities.

## Discussion

Our results show a substantial correlation between CFF and decoding abilities within a “normal” (non-dyslexic) population of college students. The correlations between CFF and word and nonsense-word measures were remarkably similar, despite the fact that nonsense-word scores were markedly lower than the word scores. This makes sense considering that we were not tallying simple pronunciation mistakes, but were noting only those errors that related in word decoding. Because words that are known are not decoded, normal readers recognize simple words almost as pictures and only start decoding later in the list when the more difficult words are presented. However, with the nonsense words, individuals must begin decoding earlier in the list, and although they tend to make errors at the same level of decoding difficulty as the word measure (as exemplified by the correlations), that level of difficulty happens much earlier in the nonsense-word condition. Interestingly, high correlations were found for the non-linguistic as well as the linguistic measures of decoding, implying that the same mechanism may be involved even in the absence of linguistic processing.

The relationship between CFF and decoding is consistent with recent data showing a role of dorsal stream processing in object recognition. For example neuro-imaging research exploring top-down processing in object recognition, suggests that the magnocellular system may facilitate object recognition through the orbitofrontal cortex [27]-[29] by providing a low-resolution view of an object (i.e., a gist) that facilitates ventral stream processing. It may be that unknown words have their constituent parts processed as objects in much the same way that one would differentiate any ambiguous object, not as a linguistic unit, but as a group of base elements that needs to be identified and then grouped for linguistic decoding.

This is consistent with the role of the dorsal pathway in dyslexia [22], [30], [31]. Motion processing has long been regarded as a correlate of dorsal stream function [32], [17], [33], [20], and the ability to resolve visual modulation (flicker) is believed to be limited by the dorsal stream within the primary visual cortex [34]. Given that reading involves rapid eye-movements and fast processing of visual information, numerous researchers have suggested that the dorsal stream is critical for the visual processing involved in determining reading abilities. Consistent with this finding and our own results, Liederman et al. [35] found that inhibiting the V5/MT+ region of the visual system through transcranial stimulation disrupted the participant's ability to read nonsense words.

Considering that there is evidence of considerable crosstalk between the dorsal and ventral streams [36], it makes sense that dorsal input can facilitate some ventral activities. Additionally, Au and Lovegrove [37] found evidence that both rapid visual and auditory processing contributed to reading irregular words and pseudowords. Moreover, a recent study conducted by Cohen, Dehaene, Vinckier, Jobert, and Montavont [38], which employed both behavioral and neuro-imaging techniques, concluded that there are likely two systems involved in reading: A ventral word-form recognition system, used for normal reading; and a dorsal system that is deployed when the reading task is serial and demanding, such as a child learning new words or a normal reader deciphering nonsense words.

We propose that a fruitful direction for future work would be to build upon the correlations found in the current study to explore whether causal links exist between dorsal stream processes and reading. In particular, whether a perceptual learning paradigm that is known to increase temporal processing will benefit individuals who have diminished CFF thresholds, such as those who suffer from dyslexia and related reading disabilities. Given previous research [39], [40], it seems likely, that we can increase CFF thresholds in patient populations that have a diminished capacity to process flicker (e.g., dyslexics). If so, it is possible that we may be able to alleviate some of the symptoms of reading disability. It is important to note that it is unlikely that a perceptual learning paradigm could improve reading ability in a severely disabled reader without other interventions. However, a person with reading disabilities might have less difficulty learning to read if an intervention, applied prior to or in conjunction with a reading program, was applied to strengthen basic visual processing.
